# Crumbs: Lightweight Daily Food Challenges to Promote Engagement and Mindfulness

**DOI:** 10.1145/2858036.2858044

**Published:** 2016-05

**Authors:** Daniel A. Epstein, Felicia Cordeiro, James Fogarty, Gary Hsieh, Sean A. Munson

**Affiliations:** 1 Computer Science & Engineering, DUB Group, University of Washington; 2 Human Centered Design & Engineering, DUB Group, University of Washington

**Keywords:** Food, daily challenges, mindfulness, personal informatics

## Abstract

Many people struggle with efforts to make healthy behavior changes, such as healthy eating. Several existing approaches promote healthy eating, but present high barriers and yield limited engagement. As a lightweight alternative approach to promoting mindful eating, we introduce and examine *crumbs*: daily food challenges completed by consuming one food that meets the challenge. We examine crumbs through developing and deploying the iPhone application *Food4Thought*. In a 3-week field study with 61 participants, crumbs supported engagement and mindfulness while offering opportunities to learn about food. Our 2×2 study compared *nutrition* versus *non-nutrition* crumbs coupled with *social* versus *non-social* features. *Nutrition* crumbs often felt more purposeful to participants, but *non-nutrition* crumbs increased mindfulness more than *nutrition* crumbs. *Social* features helped sustain engagement and were important for engagement with *non-nutrition* crumbs. *Social* features also enabled learning about the variety of foods other people use to meet a challenge.

## Introduction

Daily interventions such as reminders, tips, or challenges, are a popular and effective method for promoting a variety of aspects of well-being [11,35]. Challenges provide small, achievable goals that give a clear indication of whether they have been completed, and can support competition or fun. Daily challenges have been shown to promote behavior change [11] and provide feedback on efforts toward larger goals [12].

Mindfulness focuses attention on the present activities [23] and can lead to healthier behaviors. In our focus on food, we build upon a definition of mindfulness as “*non-judgmental awareness of physical and emotional sensations associated with eating*” [19]. Mindfulness is therefore explicitly not corrective, does not make behavior change recommendations, and does not judge food “good” or “bad.” Food mindfulness can help people understand why they eat [19,42], cope with cravings [1], avoid eating disorders [5,24], and manage obesity [24,34]. Successful methods for promoting food mindfulness have included hosting information sessions and discussions [5,24] and providing people with manuals [1]. Other common food tracking techniques, such as journaling, can also promote mindfulness of food choices [6,10,14,27,41]. However, people find food journals difficult and burdensome, often feel judged by tracking tools, and report abandoning their efforts due to these feelings of judgment [14,15].

We draw on research on daily challenges [35,41] and photo-based food journaling [6,14] to develop the concept of *crumbs:* lightweight food-based daily challenges. A crumb is a daily challenge that a person completes by taking a picture of and consuming one food or meal that meets the challenge requirement. We design and evaluate four variants of an experience using crumbs: examining the role of a *social* experience in daily food challenges and the *nutritional* nature of those challenges.

We also incorporate successful social features found in other self-tracking domains, such as leaderboards and competition [12,40] or forums [37] in physical activity. We assess the value of social features in our current approach by comparing a personal version of the crumbs intervention with a version in which people post pictures of completed crumbs to a shared Facebook group. Although prior work suggests social experiences can help *or* hurt participation [15,17,30,31], we hypothesize that providing a social experience among people completing the same crumb can help people stay engaged with the intervention.

Noting arguments in research for both prescriptive and open-ended wellness applications, we develop and evaluate two types of crumbs. A *nutrition* crumb is nutritionally prescriptive (e.g., “Eat something high in fiber”). In contrast, *non-nutrition* crumbs are prescriptive, but not about the nutritional content (e.g., “Eat something that reminds you of your childhood”). We hypothesize non-nutrition crumbs can provide mindfulness benefits while potentially embracing the celebratory nature of food [22] and not making people feel as judged about nutritional choices and intake [14,15].

These features are combined into an iPhone application, *Food4Thought*. We evaluate our designs in a three-week field experiment with 61 participants, divided into a 2×2 design comparing *social* vs. *non-social* interventions and *nutrition* vs. *non-nutrition* crumbs. We evaluate participant engagement, mindfulness, and changes in foods consumed.

We found that crumbs generally supported mindfulness and offered opportunities to learn about food. Participants who received nutrition crumbs reported they more often learned about the nutritional makeup of their food, but participants who received non-nutritional crumbs reported larger gains in eating mindfulness. *Social* features helped sustain engagement, were particularly important to engagement with non-nutrition crumbs, and created an opportunity to learn about the variety of foods people use to complete a challenge.

The primary contributions of this work include:

- We introduce and evaluate the concept of a crumb: a lightweight food-related daily challenge.
- Our results offer important insights into creating effective daily food challenges. A more nutritionally prescriptive challenge may result in people learning more about their nutrition, while a more creative and fun challenge may be more effective in improving mindfulness.
- We build upon existing research on the benefits of social features in self-tracking apps. We demonstrate the benefit of social features in supporting engagement, highlighting how these features increase the sense of motivation and accountability while also providing opportunities to learn from other participants.

## Background and Motivation

Many people seek to improve their diet in some way, and many approaches have been developed to help people in this general goal. These include more prescriptive techniques (e.g., following a specific diet or reaching certain goals for nutritional content) as well more open-ended techniques (e.g., mindfulness exercises or journaling food eaten without other specific requirements about what is eaten). Regardless of technique, awareness of food and the act of eating is typically a key component of the intervention.

In developing and evaluating crumbs and Food4Thought, we were motivated by research on the benefits and challenges of food journaling, the benefits of increased food mindfulness, and the potential of daily goals and social features to promote engagement. We now review this literature.

### Food Journaling

Food journaling is one of the most commonly recommended techniques for gaining awareness of food eaten and for self-regulation of diet. Early food journaling methods used lengthy interviews and questionnaires to collect an understanding of patient eating habits [28]. Paper diaries were later introduced, assisting recall by allowing people to record food nearer to the time of eating. Mobile food tracking applications have more recently become available, and many people prefer them to paper diaries [8].

Despite the prevalence of journaling, food journalers often find it tedious to keep an accurate record of all food consumed, and often fall out of the habit [15]. To reduce the burden, research has examined tracking only meal components (e.g., vegetables, grains) [3], taking meal photos [6,10,14], or marking a meal as “healthy” or not [29]. Byrne et al. demonstrated the potential for an intervention to change eating habits based on one photo of food per day. Adolescents used their app to take a photo of their breakfast, and then used these photos to take care of a virtual pet [10].

People can also give up journaling food or become frustrated with it because it makes them feel judged. In particular, the emphasis on quantification and calorie budgets in many journals can also create feelings of judgment that lead people to abandon journaling [14,15].

### Eating Mindfulness

Mindful eating interventions are another technique that also focuses on attention and awareness [23]. Mindfulness focuses attention on present experiences without judgment and without saying what the experience should be [19,23]. Mindfulness interventions are not based in “good” or “bad”, nor in prescriptive behavior change. Framson et al. define eating mindfulness as “*non-judgmental awareness of physical and emotional sensations associated with eating*” [19]. Eating mindfulness practices have been found to decrease cravings [1] and reduce BMI for those overweight [24,34].

Many mindful eating programs have taken a Cognitive Behavioral Therapy approach to helping people identify and adopt healthier habits. These often involve in-person sessions [24] or daily home mindfulness exercises [2], spanning multiple months. These mindfulness interventions are designed to mitigate serious eating disorders, and are fairly time-consuming and demanding of participants.

### Supporting Engagement & Reducing Barriers in Tracking

In this research, we strive to address two barriers people encounter when engaging with common food interventions. To do so, we design an intervention that imposes lower burdens and that people perceive as less judgmental.

To address our first goal, we draw upon research in small, daily challenges and upon research in social engagement.

#### Engaging with Small, Daily Challenges

Setting small, achievable goals is a key strategy for behavior change (i.e., take small steps [18]). Goal-setting theory further suggests people exert the most effort when given specific goals [26]. Concrete challenges remove ambiguity about whether and when goals are attained. Publicizing completion of challenges enables public celebration of achievement, which further increases its salience [13].

The use of daily goals has been explored in various research projects and commercial products. Many physical activity applications suggest daily goals (e.g., Houston [12], FitBit). These daily goals tend to be basic and repetitive (e.g., number of steps or active minutes per day). Such goals are effective when tracking requires little effort and feedback is persistent [12], but more varied and interesting goals may support greater engagement and learning opportunities over time.

The Daily Challenge program [11] is one example of a wellness program based on varied, small goals. This program presented participants with a challenge each day, such as reading about where they should store fruit, taking the stairs at work, learning about the salt content of their lunch, or learning about a local issue. In a 30-day trial, participants in this program had greater improvements in well-being scores than participants who received an e-health newsletter [11].

Importantly, we did not design crumbs to replace journaling of all foods for people working to achieve a specific nutrition budget. Crumbs require people engage in only one daily challenge, which consists of photographing just one food selection that meets the challenge's requirements. Informed by results on daily challenges [11] and Byrne et al.'s success with using daily food photos to motivate behavior change in youth, we anticipated that this design of crumbs could present lower barriers but still be sufficient to result in benefits for people generally seeking to improve their eating.

#### Social Features and Engagement

Prior research also examines social features for increasing engagement in technology-mediated health and wellness interventions. Social features have been used to increase emotional support, informational support, accountability, and motivation [17]. These include sharing goals and progress with one's social network [16,30,31] or on peer support communities (e.g., [6,12], FitBit) and providing opportunities to discuss barriers and ask questions in support forums [37]. Results have been mixed: social features sometimes lead to greater engagement or participation (e.g., [37]), but sometimes have no effect because participants are reluctant to use social features (e.g., [32]), or sometimes the effects cancel each other out (e.g., [31]).

Grouping people who have a common goal has been shown to motivate participation in mobile-social health applications [7]. Other studies have placed people in groups with shared goals as a way to create a shared experience, sense of togetherness, and sometimes competition (e.g., [25]). The Daily Challenge website allows participants to form social ties with other participants, and then to see who among their connections has completed each challenge. Researchers found participants who were socially connected completed 1.85 times as many challenges as those without social ties [35]. However, participants were not randomized into social or non-social conditions, so it was impossible to determine if the social connections increased engagement or if the increased engagement and social connections were instead both a consequence of higher interest in the activity. In this study, we test that casual connection.

#### Prescriptive vs. Non-Prescriptive

To address the second barrier (i.e., that interventions can be perceived as judgmental, contributing to abandonment), we build upon prior research suggesting activities may not need to be prescriptive to promote mindfulness. The designers of VERA argue that open-ended systems which encourage reflection can offer flexibility that may appeal to people [6]. Grimes and Harper argue the design space for health and wellness systems should include celebratory technology, incorporating positive interactions people have with food to allow room for creativity and pleasure [22]. O'Hara et al. describe using photo mementos of meals as one such technology [33]. Instead of limiting daily goals to nutrition-based or health-oriented challenges (e.g., “eat 100 grams of fiber”), it may be just as beneficial to offer more fun, open-ended challenges (e.g., “eat something purple”). As a result, we designed and evaluated both nutritionally prescriptive and non-nutritionally prescriptive crumbs.

### Research Questions

We designed a study to explore and evaluate different points in the design space for lightweight daily food challenges. We sought to design engaging food tracking experiences that would improve eating mindfulness and provide opportunities to learn about food. We selected eating mindfulness as an outcome because it can support many healthy eating goals and is not tied to any single food outcome (e.g., achieving a calorie budget). It can therefore benefit a broad range of people.

We specifically investigated the following research questions:

RQ1. Do crumbs (i.e., lightweight daily food challenges), offer an engaging food tracking experience that results in food mindfulness and learning?

We hypothesized that crumbs can support an engaging experience and would benefit mindfulness and learning.

RQ2. Does (a) engagement or (b) mindfulness differ whether participants receive nutritionally prescriptive challenges or challenges that are not nutritionally prescriptive?

We had competing hypotheses. Non-nutrition crumbs might be more fun and engaging, as they are less prescriptive and allow participants to reflect on or celebrate their behaviors, leading to greater mindfulness. On the other hand, nutrition crumbs may promote greater consideration of the health consequences of food choices and thus greater mindfulness. We also believed nutrition crumbs might provoke additional opportunities to learn about the nutritional makeup of foods, often considered a benefit of traditional food journal methods.

RQ3. Does a discussion board supporting conversation about challenges and sharing challenge achievement with other participants (a) change engagement in the challenges or (b) result in different mindfulness outcomes?

We hypothesized social features would lead to greater engagement and thus greater mindfulness. We further believed social features would provide opportunities for participants to learn from each other.

## System Design

To evaluate crumbs as tools for engagement, mindfulness, and learning, we developed the mobile food journaling app Food4Thought. Rather than requiring tedious tracking of all foods, it encourages journaling one food or meal a day that meets a daily challenge. The system has two components: (1) a mobile photo-based food journaling app, and (2) a private Facebook group to which photos meeting the day's challenge are posted. We selected Facebook as the social platform because it incorporates the app into many people's routines. It offers methods for encouragement (e.g., likes, comments) and communication (e.g., comments). People also see Food4Thought posts when accessing Facebook for other reasons. Because the Facebook group was private, posts were only visible to others in the study, creating a cohort effect similar to other systems (e.g., [6,7,12,25,35], FitBit).

**Figure 1:**
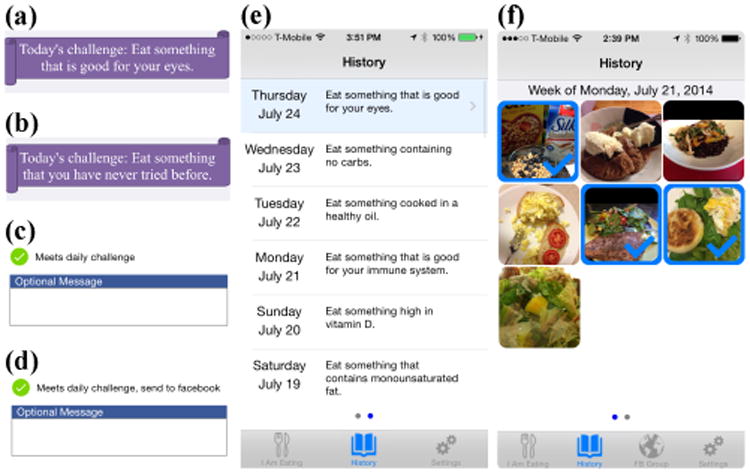
Food4Thought is a mobile food journaling app providing daily challenges and a historical log of food photos. We developed four versions based on a 2×2 design comparing (a) nutrition and (b) non-nutrition crumbs, and (c) non-social and (d) social interventions. Food4Thought included looking back at (e) completed challenges and (f) all pictures taken.

The mobile app displays a notification of the day's crumb at a configurable time (default 9am), a lesson learned from other journaling systems [9]. A person completes a challenge by taking a picture of a food they ate (Figure 1a, 1b), indicating whether it meets the day's challenge, and adding an optional message. A person can look back at the previous day's challenges (Figure 1e) and at all pictures they have taken with the app (Figure 1f). If an entry meets the challenge, the picture and message are sent to the Facebook group (Figure 1d). Photos are posted to a separate album for each day's challenge, and each photo appears in the group's feed. At midnight each day, Food4Thought automatically posts the number of people who completed the previous day's challenge and a link to the corresponding album. This allows seeing an overview of food others ate to complete the challenges, which later sections will discuss as an important element of the design.

## Crumb Development and Selection

We define a crumb as a food-related challenge completed by consuming *one* food or meal that meets the challenge. Crumbs begin with a statement of “Eat something …” and end with an attribute of food (e.g., “Eat something crunchy”). To accomplish a crumb, a person must eat a food with that characteristic by the end of the day. This small, actionable challenge requires additional thought about food consumed to complete it, and could therefore increase mindfulness. Crumbs also provide opportunities for food journaling to be fun, social, or competitive while supporting learning.

### Nutritionally and Non-Nutritionally Prescriptive Crumbs

We designed and studied two types of crumbs: nutritionally prescriptive and non-nutritionally prescriptive. A nutrition crumb is a challenge that requires thinking about the nutritional makeup of food (e.g., the amount of protein, low sugar, high fiber). A specific example is “Eat something high in vitamin B-12.” We chose this type of crumb to be consistent with nutritional information tracked in current food journaling applications.

Inspired by prior work [6,22], we designed non-nutritionally prescriptive crumbs to promote a less corrective and more creative approach to healthy eating. A non-nutrition crumb emphasizes general food attributes (e.g., color or shape, the letter a food begins with, a food's preparation). A specific example is “Eat something that is triangular.” Non-nutrition crumbs may imply nutritional values (e.g., “Eat something cooked in a microwave”, “Eat something that is a dessert”), but are not nutritionally prescriptive. A person completing these crumbs may still make nutritionally beneficial choices (e.g., reheated roasted vegetables, nonfat yogurt), although it is not required (e.g., a pizza roll, an ice cream sundae).

Two members of the research team generated 202 total crumbs. We found it easier to ideate for non-nutritionally prescriptive crumbs, and so we unintentionally generated unequal numbers of the two categories: 42 nutrition and 160 non-nutrition. The supplementary materials contain a full list of crumbs generated.

### Verification of Crumb Categories

People have varying beliefs about nutrition, and validity of our experiment required the crumbs in each category reliably be considered as nutritionally prescriptive or non-nutritionally prescriptive. To verify and categorize our crumbs, we obtained opinions from people on Amazon Mechanical Turk. We required Turkers have a 90%+ HIT acceptance rate and at least 100 HITs approved. Given food's cultural nature, we required all Turkers be from the United States to ensure some similarity in food background. Each HIT required rating five different crumbs presented on a single page. We paid Turkers $0.15 per HIT. Turkers rated each crumb according to their agreement with several statements on 5-item Likert scales. Statements included: (1) “I could easily complete this challenge”, (2) “This challenge would be difficult to complete”, and (3) “This challenge requires me to think about the nutritional makeup of food.”

To identify spam responses, we asked both (1) “easily complete” and (2) “difficult to complete” as questions about opposites, to filter responses from Turkers who responded similarly to both questions. We removed all responses where Turkers agreed or disagreed with both questions (i.e., on the same side of a 3 rating on our 5-item Likert scale, labeled as “neutral”). We also removed all responses from a Turker if they gave the same Likert value across all statements for five or more crumbs. In total, we removed 7.84% of responses as spam. After spam removal, an average of 14.3 Turkers (min: 9, max: 19) evaluated each crumb.

#### Filtering and Categorizing Crumbs

Before categorizing crumbs as nutrition or non-nutrition, we filtered out potentially controversial crumbs to ensure a clear distinction. For a crumb to be controversial: (1) the median “nutritional makeup” rating from all Turkers must be a neutral 3, or (2) the interquartile range must be greater than two, indicating high variance in Turker responses. We categorized 42 crumbs as controversial (8 by first criterion, 34 by the second, totaling 20.8%). We did not use these crumbs in our field study of Food4Thought.

Remaining crumbs were grouped by median rating of their “nutritional makeup.” Crumbs with a median greater than 3 were grouped as “nutrition” (53 crumbs) and the remaining as “non-nutrition” (107 crumbs). For 149 (93%) crumbs, Turkers agreed with the research team's initial assessment. The remaining 11 (7%) were categorized differently (“vegan”, “vegetarian”, “organic”, “fresh and in-season”, “grain,” “starchy,” “colorless,” “salty,” “citrus,” “you would eat before running,” “cooked in a healthy oil”). Turkers believed these challenges required considering the nutritional makeup of food, while the research team had not.

We believe the majority of the controversial and differently categorized crumbs arose from Turkers thinking through, and subsequently rating, which food they might eat to complete the crumb, rather than rating the crumb itself. For example, the challenge “Eat something yellow” might lead a person to think of a banana. If they consider this a nutritional fruit, they may deem the challenge to be nutritional. However, the challenge does not prescribe something healthy and could also be met with a lemon tart.

## Field Study of Lightweight Daily Food Challenges

We conducted a 3-week field deployment of Food4Thought with 61 participants, split in four groups in a 2×2 design: receiving *nutrition* (N^+^) or *non-nutrition* crumbs (N^−^), and being in a Facebook group *social* (S^+^) or a *non-social* (S^−^) condition. Participant demographics appear in Table 1.

### Participants and Crumb Selection

We recruited from Facebook, Twitter, and university and local mailing lists, describing our study as “using a food application with a random daily challenge.” Participants needed to have a Facebook account, own and use an iPhone, and remain in North America during the study (to keep similar time zones within the Facebook group and to ensure similar food opportunities).

We stratified participants by Facebook use, ensuring each social condition had equal proportions of people who regularly posted to and read their Facebook timelines. Three people had limited Internet access, so we placed them in the non-social groups such that unreliable Facebook access would not weaken the social intervention. Beyond this, condition assignment was made randomly. Groups contained unequal numbers of participants to ensure social groups had critical mass. Groups were assigned during recruitment, and some participants did not complete recruitment. Six did not start the study after recruitment (4 S^+^N^+^, 1 S^+^N^−^, 1 S^−^N^+^): three did not install Food4Thought, two did not log in, and one dropped out after learning she was pregnant. This participant reported that food photos in her Facebook feed were incompatible with her pregnancy-related nausea.

Using the results from the Mechanical Turk evaluation, we selected challenges to integrate into Food4Thought based on our split of nutrition and non-nutrition crumbs. Within the split, challenges were randomly selected (Table 2).

### Procedure

The study consisted of a pre-study survey, a three-week field deployment, a post-study survey, and an optional post-study interview. Participants installed Food4Thought on their own phones. They were compensated $30 and required only to complete the surveys and leave Food4Thought installed for the duration of the study, so challenges would appear daily. Crumb completion was not financially incentivized, and most participants did not complete all crumbs received.

The initial survey contained installation instructions, demographic questions, and questions regarding prior food journaling experience. The final survey contained multiple choice and Likert-type questions about experiences using Food4Thought. Both are available in supplementary materials.

To better understand experiences with Food4Thought and crumbs, we also interviewed 19 participants about their experiences (6 S^+^N^+^, 6 S^+^N^−^, 4 S^−^N^+^, 3 S^−^N^−^). We selected interview participants based on their responses to open-ended questions in the post-survey (we sought participants who enjoyed or disliked aspects of the study), activity in the social groups, and varied uses of the application (such as journaling all food consumed). An external service transcribed interview recordings, and the research team identified themes and representative quotes.

### Measures and Evaluation Metrics

#### Engagement

We recorded the number of food photos taken with the app (including those marked as completing daily challenges and those not). Social engagement in the social conditions was observed by reading Facebook comments and likes. Usage data was supplemented with survey and interview findings.

#### Eating Mindfulness

To evaluate the impact of Food4Thought as a mindfulness intervention, we compared scores pre- and post-intervention on the Fred Hutchinson Mindful Eating Scale, a validated scale for mindful eating behaviors [19]. The authors of the scale describe it as “*non-judgmental awareness of physical and emotional sensations associated with eating.*” This scale asks people about their observations and actions around eating, two key principles in mindfulness. In contrast to other eating scales (e.g., the emotional eating scale [4], the three-factor eating questionnaire [39], the binge-eating questionnaire [20]), it is general purpose and does not measure a particular nutritional goal or problem. It is therefore well-suited to participant goals of generally healthy eating.

Questions ask about the frequency of certain food behaviors on a 4-point scale (from Never/Rarely to Usually/Always), of how aware a person is of their food when they are eating (e.g., “*I notice when there are subtle flavors in the food I eat*”), what distracts them from eating (e.g., “*My thoughts tend to wander while I am eating*”), and external factors that contribute to them eating (e.g., “*I recognize when I'm eating and not hungry*”). We used this scale to answer RQ2b and RQ3b. We used three questions from each of the awareness, distraction, and external scales to reduce time burden of the survey. We did not include questions for scales less relevant to our research questions, specifically disinhibition (i.e., “*the inability to stop eating even when full*”) and emotional (i.e., “*eating in response to negative emotional states*”) scales.

To assist interpretation of these scales, we note that people currently completing at least two hours of yoga per week score 0.30 better on the summary score of the Mindful Eating Scale (i.e., an average of the subscores), while people who have practiced yoga for at least five years score 0.26 better than people who have never practiced yoga [19].

Participants completed this questionnaire in both pre-study and post-study surveys, and we compared change in scores. Four participants did not complete this questionnaire (one in each condition). We report on the remaining 57 participants. Across all participants, awareness and external scores were highly correlated (*r*=0.49, *p*<0.001). We combine them into a single score, and report the change in combined score and change in distraction score from before and after the study.

## Field Study Results

We present our field deployment results in terms of overall Food4Thought usage and our three research questions.

### Overall Application Usage

Over the three-week study, participants took 951 pictures of food, 551 of which they identified as completing the daily crumb (some completed the crumb multiple times per day). Participants recorded an average of 15.6 meals (min 1, max 92, average 0.7 per day), 9 of which completed crumbs. Each crumb was completed by at least four participants. Additionally, a server issue for three days potentially resulted in a small loss of data (we estimate approximately 20 entries lost in total, based on participant self-reported estimates).

72% of participants reported they enjoyed receiving crumbs and 46% wanted to continue. S^−^N^−^9 checked each day's challenge before she went to bed: “*often times I would be pretty excited about the next day's challenge and so I would look at midnight to see tomorrow's challenge*.” S^+^N^−^5 even described “*gaming the app*” by changing her phone time and date to look ahead at the next few challenges.

### Engagement and Mindfulness with Crumbs

**Figure 2:**
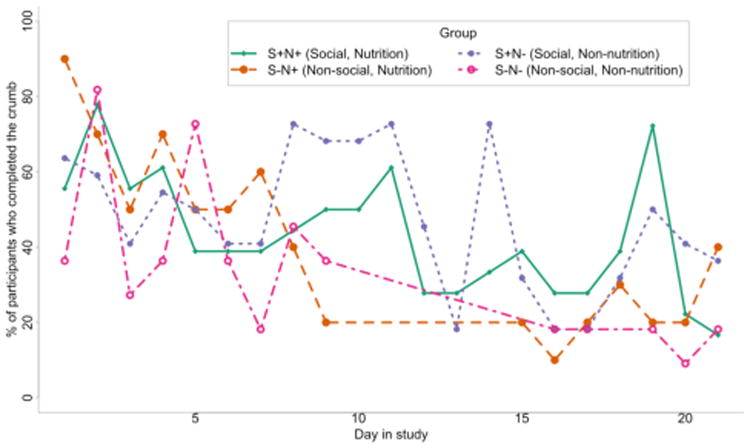
Certain challenges, particularly in the social conditions, resulted in greater completion

Figure 2 shows usage across study conditions over time. Negative binomial regression analysis with participants as a random effect (Table 3) showed a decrease in the number of challenges completed over time (Table 3 Days in Study, 95% CI: 0.8-0.9 fewer people complete a challenge per day across the study). Use of a system often decreases as its novelty wears off, and the effects of reminders also wear off over time [9,21,38]. Participants also described being more engaged at different times in the study: “*I think towards the end I got a little bit lazier*” (S^−^N^−^9), “*Towards the middle, I would get a little bit forgetful*” (S^+^N^−^13), “*at the end I got more into the rhythm of it*” (S^+^N^+^11). Reasons for less engagement included: “*a little bit forgetful*” (S^+^N^−^13), “*other things going on that just had my attention*” (S^+^N^+^11), “*vacation*” (S^+^N^−^5), and “*a new job*” (S^−^N^−^9).

During post-study interviews, participants also remarked that Food4Thought increased mindfulness. S^+^N^+^17 said “*As it is, I've been trying to eat healthier, but it's definitely made me more aware”* and that the crumb was on her mind throughout the day “*I always had it on my mind throughout the day, so I definitely think it helped.*” 37 (61%) participants agreed or strongly agreed challenges made them think about food choices for the day. Overall, nutrition and non-nutrition crumbs both prompted participants to think about their food choices for the day, supporting our hypotheses for RQ1.

#### Changing Food Choices

In both the pre-study and post-study surveys, we asked participants to estimate how many servings of fruits and vegetables and how many servings of high-fat foods (e.g., red meat) they consumed over the past seven days. Participants across all conditions reported increasing their fruit and vegetable consumption since using the application (t_55_=1.74, *p*<0.05) but leaving their high-fat consumption unchanged (t_55_=-0.68, *p*≈0.75). While we cannot distinguish whether participants changed eating behaviors as a result of the application or overall study participation, this is a promising indicator that crumbs may lead to behavior change.

In the surveys and interviews, many participants said they changed what they ate to complete a challenge: “*There was one day, eat something that has an A and I ate applesauce for breakfast instead of some other fruit. The same thing with ‘eat something sour’. I ate yogurt for breakfast, for that reason*” (S^+^N^−^9) and “*one of the ones that was high in Omega 3, so I decided to have fish for dinner instead of whatever I was going to ordinarily have*” (S^−^N^+^9).

In some cases, participants changed what they ate for the better: “*I know oranges are very hard to peel, they're kind of annoying, they make your hands dirty, but I know I probably should be eating them, so that one I ate*” (S^−^N^+^5). However, some changes were for the worse: “*there were days like, ‘Eat something from a package.’ That was actually the first day of my vacation… we had just filled our kitchen with amazing beautiful fruits and vegetables, and I would not have eaten anything from a package that day, at all, if I hadn't had that challenge*” (S^+^N^−^5).

#### Additional Journaling

Although we designed Food4Thought for daily completion of crumbs, people could also use it to keep a photo-based journal of other food. We define ‘journaling behavior’ as logging food that does not complete a crumb. Although participants in social conditions completed more crumbs than participants in non-social conditions, participants in non-social conditions created slightly more journal entries (Table 4b Social, 95% CI 0.05 to 0.11 more foods during the study). We believe participants in social conditions viewed participation in social challenges as the primary purpose of the app, while some participants in the non-social conditions saw Food4Thought as a food journaling application with the challenges as supplemental feature (e.g., S^−^N^+^8 described them as “*a nice added bonus*”).

### Differences between Nutrition and Non-Nutrition Crumbs

**Figure 3:**
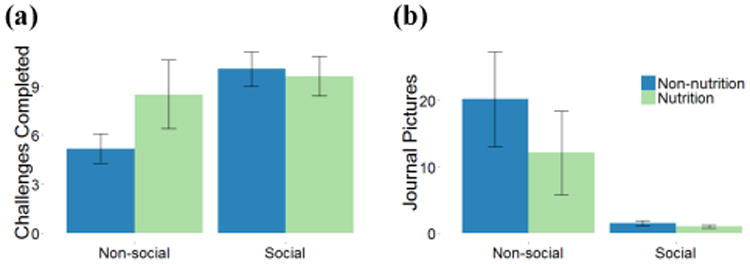
Observed interaction effects between conditions for (a) challenges completed and (b) journaling behavior. Error bars represent 95% confidence intervals.

We used negative binomial regression analysis to characterize correlations between application usage and study condition. Results are summarized in Table 4, with interaction effects summarized in Figure 3. Table 5 summarizes the difference between food mindfulness scores pre-study and post-study.

#### Engagement (RQ2a)

Participants who received nutrition crumbs completed more challenges than those who received non-nutrition crumbs (Table 4a Nutrition, 95% CI 1.2-2.3 more crumbs out of 21 during the study). Participants in nutrition crumb conditions said challenges often encouraged improvements they already wanted to make, so they were more motivated to complete them: “*‘something low in sugar’. That, in particular, just aligns with my personal dietary beliefs*” (S^−^N^+^3). People in non-nutrition conditions often felt crumbs were arbitrary or did not have a benefit, which made them less inclined to complete them: “*the arbitrariness of the challenges, like eating a food that begins with the letter ‘S’ … It didn't seem like they had a point*” (S^+^N^−^5); “*I didn't understand what I was getting out of doing the challenge*” (S^−^N^−^1). This is counter to our expectation non-nutrition crumbs would lead to higher engagement, though the next section notes social features interacted with crumb type: participants were more engaged with non-nutrition crumbs in the social condition.

#### Mindfulness (RQ2b)

We observed a negative effect of nutrition-focused crumbs on the combined awareness and external scores (Table 5a Nutrition, 95% CI: 0.26-1.85 decrease). Given the overall increase in mindfulness reported in the previous section (Table 5a Intercept, 95% CI: 0.25-1.35 increase), this means that although participation in the study contributed to awareness, it had less of an effect in the nutrition conditions. This is consistent with our hypothesis that the corrective nature of nutritionally prescriptive challenges can interfere with the non-judgmental nature of mindfulness.

#### Opportunities for Learning

Participants in nutrition and non-nutrition conditions said they looked up foods: “*For almost all of the letter ones, I Googled lists of foods that start with ‘A’, lists of foods that start with ‘Q’*” (S^+^N^−^5), “*I Googled, saw what was high in Vitamin C*” (S^−^N^+^9). Crumbs that involved looking up foods were the favorite for participants in the nutrition condition, as they found them more fun: “*the ones that required me to look something up were more fun*” (S^+^N^+^13). A few participants also said they enjoyed when crumbs made them learn about nutritional factors they did not otherwise consider: “*My favorite ones were… the Vitamin D ones and the ‘eat something low on the glycemic index,’ I guess those are two things I didn't know much about*” (S^+^N^+^17).

Participants also liked learning about new foods and their health benefits: “*I liked the ‘eat something good for your liver’ challenge because I had to look up things that were good for my liver, so I learned something new*” (S^+^N^+^3). Even when not looking up food, participants reported looking more closely at their food and its health benefits, such as S^+^N^+^8: “*it made me more conscious of what I was eating … make me more aware of, ‘Hey, what's the health benefit of my Greek yogurt’*”.

### Role and Effects of Social Features

The social intervention amplified engagement, especially for non-nutrition crumbs. Participants in the social condition also described learning from other participants.

#### Engagement (RQ3a)

Participants in the social conditions completed more crumbs than those in non-social conditions (Table 4a Social, 95% CI 1.5 to 2.6 more out of 21 during the study). Participants in the social condition maintained their interest in the study longer (there is an interaction effect between days and being in the social condition; Table 3 Social*Days in Study, 95% CI 1.05-1.17 more people complete a challenge per day in the social condition), supporting our hypothesis in RQ3a that social features promote engagement. In interviews and surveys, participants in the social condition described how the social experience increased their motivation and accountability: “*You just don’t want to be the person that doesn’t complete any challenges*” (S^+^N^−^13). S^+^N^−^3 found posts by others to be motivational, “*It was really interesting to be able to see what everyone else was doing and everyone was kind of really motivated about it… motivating me to [complete challenges].*” Some other participants in the social group became competitive with one another, wanting to complete the crumb in a better or more creative way than others: “*I wanted to do something that was a better fit for the challenge than other people did*” (S^+^N^−^9).

Social features are most important for engagement with non-nutrition crumbs. We observed an interaction between social features and whether crumbs were nutrition-focused (Figure 3, Table 4a Social*Nutrition, 95% CI 0.4 to 0.9 more non-nutrition crumbs in the social condition, in addition to the strong effect of the social condition itself). Participants in the social condition were more likely to complete non-nutrition crumbs than participants in the non-social condition. Social interaction may have given these otherwise “arbitrary” non-nutrition crumbs a purpose or meaning.

Participants in the social conditions also commented that notifications about posts by others reminded them to engage with the application. S^+^N^−^4 said “*Sometimes throughout the day I would forget and then I would see some people post stuff on Facebook I would say, ‘Oh yeah. I need to go do that’.*” These reminders, as well as the accountability and competitive aspects of the social group, likely supported the higher completion rate and lower drop-out over time.

##### Experiences with sharing

**Figure 4:**
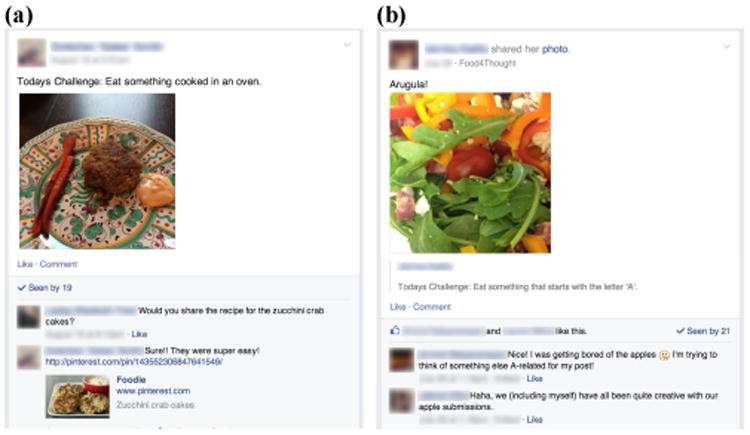
People in social conditions used Food4Thought to (a) learn from others such as by asking for a recipe, or (b) demonstrate creativity in their food choices.

Participants generally desired more social interaction. S^+^N^−^9 jokingly recommended we “*make people post more*” when asked for potential study changes. S^+^N^+^11 tried to engage socially, but was unhappy with her responses: “*I put a little comment on [a post], the guy replied and we moved on, but I was hoping for conversation*” But most felt social interaction was supportive: “*We're very supportive of others, people were asking for recipes for things that they made and stuff It was just nice. It's a group of strangers who were all doing the same thing*” (S^+^N^−^6). Participants regularly liked posts, and occasionally asked for recipes (Figure 4a). Some felt uncomfortable sharing pictures of their food with people who were not friends: “ *I felt a bit awkward initially sharing what I'm eating* (S^+^N^−^3). However, when asked if they would have completed as many challenges without the group, most agreed with S^+^N^−^13: *“I would have completed less”*.

Figure 4b shows a discussion between participants about the creativity of their responses to a challenge. Participants worried about being creative, and many often thought of the same foods to complete a challenge. For “Eat something that starts with an ‘A’”, there were several apple posts, followed by a discussion of how nice it was to for someone to complete the challenge with arugula. Seeing that others shared a picture of the same food they had planned discouraged some participants from completing a challenge: *“if someone else thought of something before me then I felt like I couldn't use that as my challenge because someone else had taken it”* (S^+^N^−^4), *“if a bunch of people had put ‘carrots’, I'm not going to put ‘carrots’*” (S^−^N^+^9).

Results support our hypothesis for RQ3a: a social experience made crumbs more engaging than individually completing them by offering motivation, accountability, and competition.

#### Mindfulness (RQ3b)

Participants in the nutrition-social condition experienced a decrease in identification of food distractions (Table 5b Social*Nutrition, 95% CI 0.09-1.41 decrease). It is possible that the additional social reminders were sometimes a distraction or that social engagement with crumbs took their attention away from the eating experience.

#### Opportunities for Learning

As in the non-social condition, participants in the social condition looked up foods that completed a crumb. Participants in the social condition further learned through discussion on their posts and by seeing others post in the Facebook group about what foods completed a crumb: *“My favorite ones were… the Vitamin D ones and the ‘eat something low on the glycemic index,’ I guess those are two things I didn't know much about”* (S^+^N^+^7). Many commented their diet was boring: *“I'm pretty boring, I tend to eat the same thing for breakfast and lunch every day”* (S^+^N^−^10). They used the Facebook group to learn about other foods: *“it was nice to see the variety of how some people completed the challenges”* (S^+^N^+^8), “ *I was looking at what others are doing and that gave me some ideas”* (S^+^N^−^3). Others asked for recipes of dishes that looked good: “ *I got a couple recipes which I'm really excited about*” (S^+^N^−^4). Participants in the social conditions preferred crumbs for which they wanted to see the responses of others, such as creative or tricky challenges: *“The homemade challenge was memorable because I enjoyed seeing what everyone else posted.”* (S^−^N^−^20)*, “the squishy one, I couldn't think of anything and enjoyed seeing what others came up with”* (S^+^N^−^2).

## Discussion

Table 6 summarizes our findings. Our overall results show the potential for low-burden and non-corrective eating interventions. People can obtain benefits in their eating, as demonstrated by the increase in reported food mindfulness, learning about food, and changes in diet, with challenges that require eating and recording only one food per day. Not only are non-nutritionally prescriptive challenges effective, they actually lead to *greater* gains in mindfulness than nutritionally prescriptive crumbs. The remainder of this section discusses challenges around engagement and re-engagement with these activities as well as future opportunities for crumbs.

### Designing for Engagement: Difficulty

We sought to design crumbs to be specific and achievable, but open-ended enough that they did not push a particular health goal and could be achieved in a variety of ways. Although participants engaged with crumbs, they still had trouble completing crumbs that were too difficult or required planning. When asked why they did not complete a crumb, 57% of participants indicated they had already decided what they would eat that day, and the challenge did not fit into their plan. Participants also sometimes had trouble identifying foods to complete a challenge (51%) or found a challenge too difficult to complete (31%). S^+^N^−^5 said *“there' like four foods that begin with the letter ‘Q.’*”

In designing the difficulty of a set of crumbs, it is important to consider that crumbs may be situationally difficult. S^+^N^+^4 said: “ *I was really bad on weekends. I left my phone places and didn't have it for long periods of time.”* Participants completed fewer challenges on weekends (Table 3 Weekend, 95% CI 0.57-0.98 fewer people), but it may be possible to design challenges more suited to weekend lifestyles. Varying difficulty to match events in a person's life might help keep a person engaged, and could support playful approaches to promoting mindfulness [21]. S^+^N^−^5 did not think the crumb itself determined the difficulty, but rather context in her life: *“the hardness felt less to do with the challenge and more to do with my life in that day.”* Designs could consider such factors as when a person plans meals, as advance notice of crumbs could inform grocery shopping. As another possible direction, a longer period could be provided for complete crumbs (e.g., “Eat something spicy this week”). S^+^N^+^10 supported this, stating: “*If I knew it sooner… I could plan to incorporate this into whatever I'm eating.*”

### Designing for Engagement: Social

Although non-nutrition crumbs promoted greater mindfulness, people were less engaged with them on their own. Based on reactions from study participants, we believe it took the social feature to give meaning to these otherwise arbitrary challenges. Future designers and researchers of mindfulness systems and other non-judgmental or non-prescriptive wellness activities might also use social features to give them meaning, or they might work to identify other techniques to create engagement around these activities.

Participants also noted opportunities for social features to be developed further. S^+^N^+^11 recommended the researchers “*cast some questions out to the group to try and stimulate some interaction between the group members*”, consistent with recommendations from prior work [36]. Another approach is to group people with similar food goals, values, or expertise to promote a shared experience, or simply to evaluate Food4Thought with a group of pre-existing friends.

Social information can also support re-engagement. Although engagement decreased as the study went on, we observed significant spikes in engagement in the social conditions (e.g., at day 13 in the non-nutrition condition: “Eat something that starts with the letter ‘Q’”, at day 19 of the nutrition condition: “Eat something that is vegan”), but no corresponding spikes in the non-social conditions. When social participants saw an influx of posts completing a challenge, they felt motivated to complete the challenge as well: “*A lot of people were posting in [the Facebook group], so I wanted to post also*” (S^+^N^−^9). Although this temporary engagement did not sustain to other days, it suggests social features paired with lightweight challenges have a potential to help re-engage people in self-tracking after a lapse.

### Future Opportunities for Crumbs

To explore the design space, we designed and selected a broad range of crumbs for inclusion in our study. Future research on crumbs might focus on understanding what aspects of those crumbs contribute to engagement and mindfulness, in both individual and social use. For example, from our study it remains unclear how specific nutrition crumbs should be. “At least 20 grams of protein” is more prescriptive than “high in protein”, and may therefore feel more judgmental.

Future crumbs, or sequences of crumbs, could be personalized to individual goals, dietary excesses, or deficiencies (e.g., a vegetarian might request crumbs that exclude meats and promote a need for other proteins). A preliminary questionnaire could identify a person's food-related goals and use them to customize a sequence of crumbs, or the survey could assess a person's food knowledge and provide challenges intended to teach them something new about food. S^−^N^+^8 recommended that challenges “*be tailored more to where I know I'm weak*.”

We also believe there is opportunity to design and evaluate sequences of crumbs for people diagnosed with a health condition with associated food requirements. For example, someone recently diagnosed with irritable bowel syndrome might participate in crumbs that help them discover substitutes for foods that commonly trigger symptoms. We imagine collaboration with nutrition experts and clinicians when designing crumbs to support these targeted goals.

## Conclusion

We developed Food4Thought to evaluate the notion of a crumb, a lightweight food-related daily challenge that people complete by taking a picture of and consuming one food or meal that meets the challenge's requirement. We also examined how nutritionally prescriptive versus non-nutritionally prescriptive crumbs and the presence of social features affect engagement and mindful eating. We did not design crumbs to replace journaling of all foods for people working to achieve a specific nutrition budget, but did show that they can provide important benefits. Nutrition and non-nutrition crumbs, whether completed individually or socially, increased mindfulness and created opportunities for learning. Although nutrition crumbs had higher participation levels and prompted people to learn more about the nutritional content of food, non-nutrition crumbs resulted in a greater increase in mindfulness. Social participation sustained engagement better than private participation, and social features supported engagement with non-nutrition crumbs. Our findings show the potential of these lightweight, even non-nutritionally prescriptive interventions, and we urge further design and study of such approaches.

## Supplementary Material

Application use guide: non-social

Application use guide: social

Notes about supplementals

Post-survey

Pre-survey

## Figures and Tables

**Table 1:** Our field study had four conditions, with participants randomly assigned, weighted toward the social conditions.

	Nutrition		Non-Nutrition	
**Social**	S^+^N^+^	N=18: 5M, 13F Age: avg 30.17, min 22, max 59	S^+^N^−^	N=22: 4M, 18F Age: avg 27.64, min 18, max 65
**Non-Social**	S^−^N^+^	N=10: 2M, 8F Age: avg 26.78, min 19, max 36	S^−^N^−^	N=1 1: 2M, 9F Age: avg 27.20, min 18, max 40

**Table 2:** Participants received daily crumbs for 3 weeks, and were assigned to nutrition or non-nutrition conditions.

Day	Nutrition Crumbs (Eat something…)	Non-nutrition Crumbs (Eat something…)
**1**	high in fiber.	that is homemade.
**2**	low in sugar.	that starts with the letter ‘A’.
**3**	high in vitamin C.	cooked on a skillet.
**4**	under 4 grams of saturated fat.	that is spicy.
**5**	low in sodium.	that is yellow.
**6**	with at least 20 grams of protein.	that reminds you of your teenage years.
**7**	that is organic.	that you would eat in the spring.
**8**	low in carbs.	that starts with the letter ‘S’.
**9**	that is salty.	that is a dessert.
**10**	that is good for your eyes.	that is squishy.
**11**	that you would eat before running.	that is room-temperature.
**12**	that is whole-grain.	that you would eat on a picnic.
**13**	high in potassium.	that starts with the letter ‘Q’.
**14**	high in Omega-3.	from a package.
**15**	that is dairy-free.	that is sour.
**16**	that is colorless.	traditionally French.
**17**	that is good for your liver.	that is blue.
**18**	low on the glycemic index.	that you would eat on Halloween.
**19**	that is vegan.	that starts with the letter ‘W’.
**20**	high in vitamin A.	cooked in an oven.
**21**	that is good for your hair.	that you have never tried before.

**Table 3:** Challenge completion rate decreased over the study. The effect was mitigated in the social conditions.

	Count Model Coefficients		
Variable	Estimate	Std. Error	p
**(Intercept)**	0.472	0.397	0.584
**Social**	0.028	0.468	0.371
**Nutrition**	0.122	0.517	0.204
Social*Nutrition	-0.837	0.586	0.153
**Weekend**	-0.321	0.152	0.035 *
**Days in Study**	-0.161	0.026	<0.001 ***
Social*Days in Study	0.102	0.026	<0.001 ***
Nutrition*Days in Study	-0.005	0.023	0.821

*** p<0.001

** p<0.01

* p< 0.05.

p< 0.1

**Table 4:** Regression results for (a) Number of challenges completed and (b) Number of pictures taken in support of a food journal, varied by level of social and nutrition variables. The intercept indicates a baseline level, while other variables denote the influence of the study conditions. Social*Nutrition denotes the influence of both conditions (S^+^N^+^) compared to when one condition was absent (S^+^N), (S N^+^).

(a) Challenges Completed			
	Count Model Coefficients		
Variable	Estimate	Std. Error	p
**(Intercept)**	1.645	0.133	<0.001 ***
**Social**	0.662	0.149	<0.001 ***
**Nutrition**	0.495	0.171	0.004 **
Social*Nutrition	-0.539	0.199	0.007 **
(b) Journal Pictures			
**(Intercept)**	3.005	0.067	<0.001 ***
**Social**	-2.599	0.187	<0.001 ***
**Nutrition**	-0.528	0.113	<0.001 ***
Social*Nutrition	0.005	0.314	0.735

*** p<0.001

** p<0.01

* p< 0.05.

p< 0.1

**Table 5:** Across Food4Thought, we find (a) improvements in food awareness, particularly in non-nutrition conditions, and (b) declines in identification of food distractions.

(a) Awareness & External			
	Linear Model Coefficients		
Variable	Estimate	Std. Error	p
**(Intercept)**	0.796	0.274	0.005 **
**Social**	-0.662	0.336	0.054.
**Nutrition**	-1.055	0.398	0.011 *
Social*Nutrition	0.920	0.490	0.066.
(b) Distraction			
**(Intercept)**	-0.334	0.184	0.076.
**Social**	0.451	0.226	0.051.
**Nutrition**	0.481	0.268	0.078.
Social*Nutrition	-0.753	0.330	0.026 *

*** p<0.001

** p<0.01

* p< 0.05.

p< 0.1

**Table 6:** Crumbs increased mindfulness and created opportunities for learning in an engaging and low-burden manner.

Questio	Measure	Findings
RQ1 (crumbs)	Engagement	Participants completed an average of 9 crumbs. 72% enjoyed receiving crumbs, 46% wanted to continue receiving them. Challenge completion decreased over time (95% CI: 0.8 to 0.9 fewer people per day).
	Mindfulness	Increase in combined awareness and external scores (95% CI: 0.5 to 1.7), decrease in food distraction score (95% CI: 0.0 to 0.8).
RQ2 (nutrition features)	Engagement	Participants completed more crumbs in nutrition conditions than non-nutrition (95% CI: 1.2 to 2.3). Nutrition crumbs better reflected people's food beliefs and felt less arbitrary.
	Mindfulness	Decrease in combined awareness and external score for participants in nutrition conditions (95% CI: 0.1 to 1.7). Non-nutritionally prescriptive crumbs promoted more mindfulness.
RQ3 (social features)	Engagement	Participants completed more crumbs in social conditions than non-social (95% CI: 1.5 to 2.6). Social condition participants did not want to let others in their group down, and found social interaction supportive.
	Mindfulness	No significant effects on the Mindful Eating Scale. Participants in social conditions reported Facebook notifications served as an extra reminder to participate.
