# Supplementary material for: Crumbs: Lightweight Daily Food Challenges to Promote Engagement and Mindfulness
Source: Proc SIGCHI Conf Hum Factor Comput Syst. Author manuscript; Available in PMC 2017 May 12. (PMC5428072; doi:10.1145/2858036.2858044)

This was the guide for the *social* conditions of Food4Thought. In these conditions, participants were in a Facebook group with other participants. Participants in each group received the same daily challenges.

Food4Thought iPhone Application Guide

Setup

We will first guide you through logging into Food4Thought.

You should have been given a group number in the email that this document was attached to. It will be a number 1-4.

When you first open the app, you will be asked for your group number. Please select the group number that you were given in the email, and click "Done".

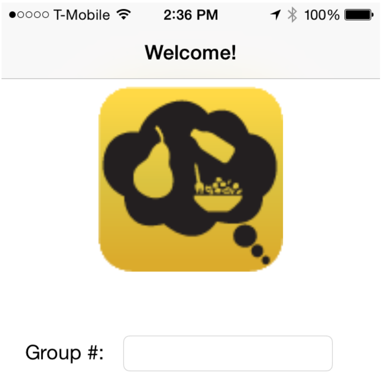

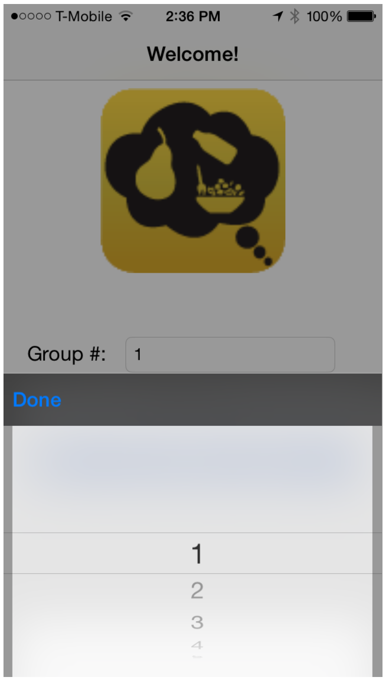

The next step is to login with Facebook. After selecting your group number, you should see a Login with Facebook button appear. Click the button and enter your Facebook username and password. Then select Log In. You may be asked to approve Food4Thought to access your Facebook information and groups. Please select OK for all questions without changing the settings (particularly who can see posts you make through the application. The app will not post publicly, but the research team needs access to posts made, so the “only me” setting is too restrictive).

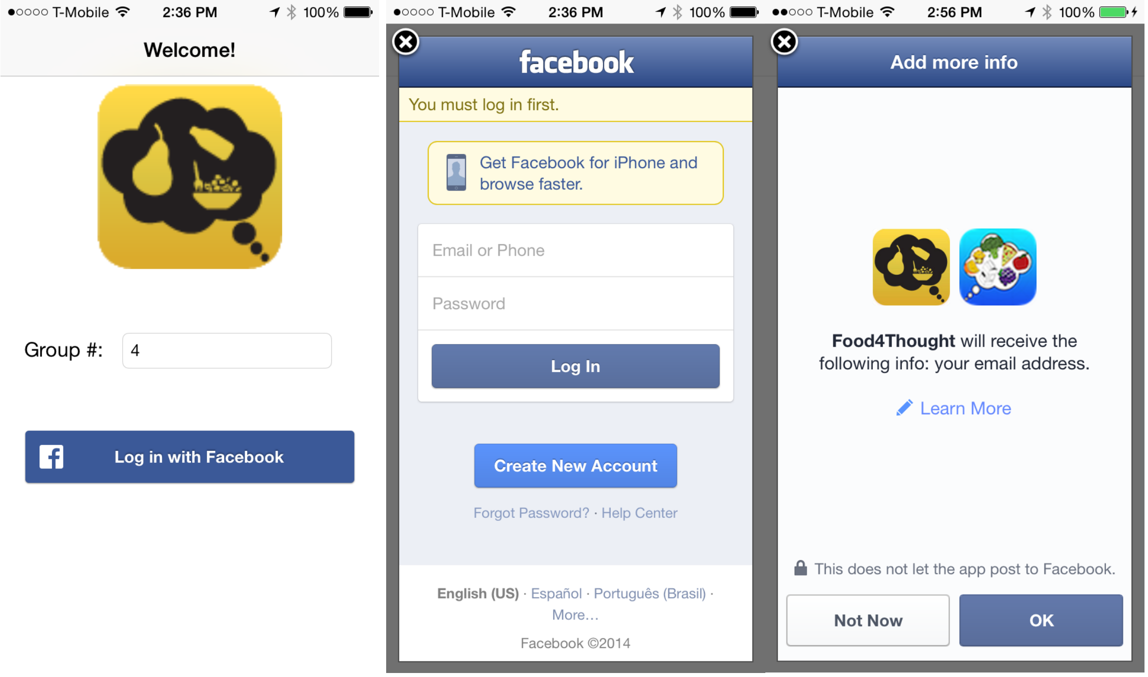

You have now successfully logged into Food4Thought! You will be asked for permission to use your current location, please select OK. While we are not recording your location, we are using it as a shortcut around how Apple resolves background applications. It allows Food4Thought to resend any food photos that were collected while the application had no network connection.

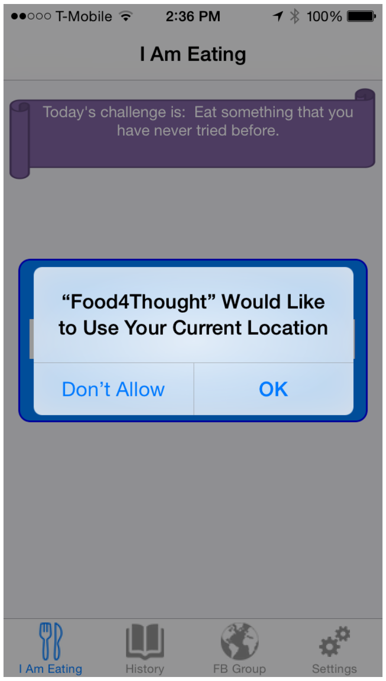

Features

We will now show you the different features of Food4Thought.

Record Food

To record food, select the “I Am Eating” tab, and then click the large blue “Record Food” button. This will pop up the camera for you to take a picture of the food you are recording.

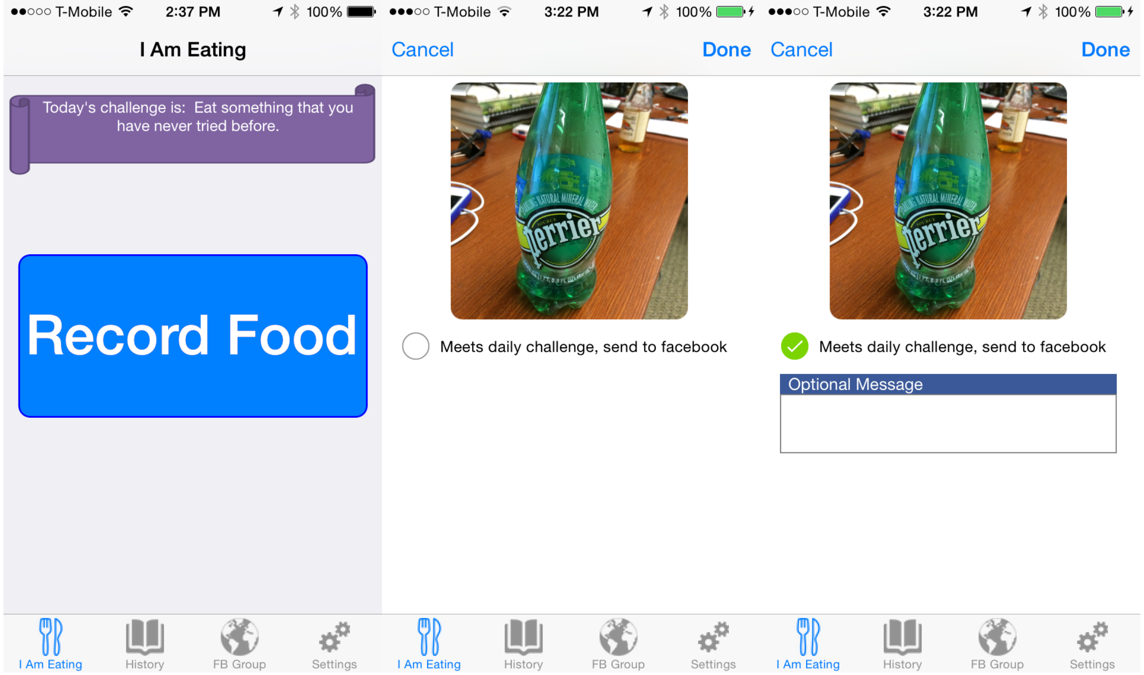

Once you take a picture of the food, you are asked if the picture completes the daily challenge. If it does, select the check box and an optional message can be added. The text in the optional message will be sent to the Facebook Food4Thought group with the photo of the food meeting the challenge. If the image does not complete the daily challenge, then do not select the checkbox.

Once complete, click “Done” in the upper right hand corner. You will then be guided to the history page.

History

To see a history of the foods that you have eaten, and the crumbs that you have completed, select the history tab. The history tab contains two pages. To switch pages, swipe left/right.

The first page shows the foods that you have taken pictures of, grouped by week. Foods that completed the day's challenge are bordered in blue and have a blue check box in the bottom left hand corner. Foods that did not complete the day's challenge will not.

The second page shows a list of challenges by day. Days that you completed the challenge will be highlighted in blue. You can select the blue rows in the table to see the food(s) that were eaten to complete that days challenge.

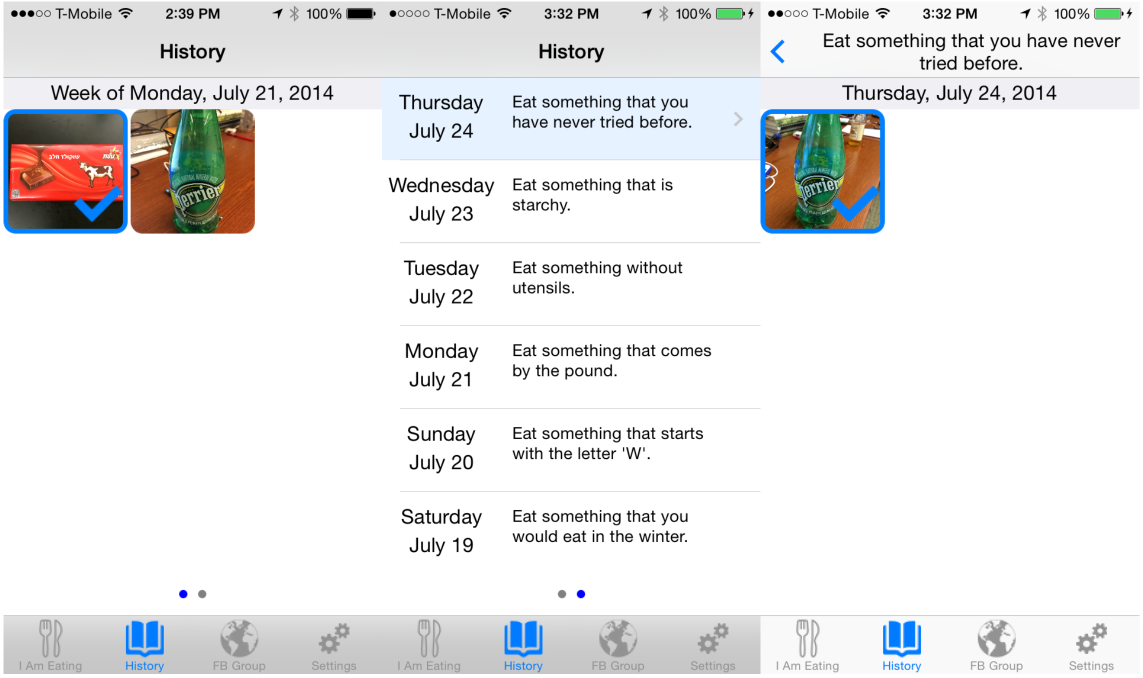

Facebook Group

The Fb group tab allows you to see the Facebook group for Food4Thought. You can also view it on your computer by selecting the Food4Thought group in the left pane under groups, or by selecting the Food4Thought group on your mobile Facebook app (available in the "more" tab in the official Facebook iPhone app).

Settings

On the settings tab, you can log out of Facebook (which you should not do during the study, but is an option to revoke our access to your Facebook at the end study). You can also change the time that you receive your daily challenge notification. The default is 9:00am, but this may not be early enough to be before breakfast for some people. Feel free to change it to whatever time suits you best.

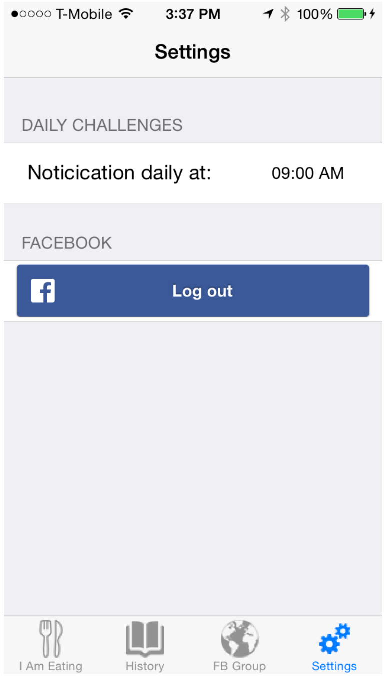

Supplement: Application use guide: social [file NIHMS855557-supplement-Application_use_guide__social.pdf]
