# Supplementary material for: Crumbs: Lightweight Daily Food Challenges to Promote Engagement and Mindfulness
Source: Proc SIGCHI Conf Hum Factor Comput Syst. Author manuscript; Available in PMC 2017 May 12. (PMC5428072; doi:10.1145/2858036.2858044)
Supplement: Post-survey [file NIHMS855557-supplement-Post-survey.pdf]

Food4Thought Final Survey

Thanks again for your involvement with the Food4Thought study! This survey will review the three weeks of the study so we can learn how you feel about the app and daily challenges.

This survey has four parts and may take **up to an hour** to complete, so please consider taking breaks.

Question 1.
Please enter your facebook ID, available in the Username field or by going to your public profile.

Question 2.
Did you encounter any technical challenges as part of the study? This could include difficulties with the app, the daily challenges, or the Facebook group (if applicable). Please describe any technical problems you had during this study.

Question 3.
What group were you assigned to for this study? If you do not remember, please contact a member of the research team.

Part 1 of 4: Daily Challenges

For each daily challenge you received, please respond by filling in the following sentence: **I found this challenge [...] to complete**. (7-item scale, very difficult to very easy)

Part 2 of 4: Challenges Completed

We will now ask you a few questions about the specific challenges you did and did not complete. Please consider taking a break before and/or after this section.

For each challenge, supplemented with pictures of the photos they took for that challenge: On a scale from 1 (strongly disagree) to 7 (strongly agree), please rate how much you agree with the following statement: I found this crumb difficult to complete.

Part 3 of 4: Memorability and Feature Usage

Select one challenge that was particularly memorable. What was it, and why was it memorable?

Question 8.
For the challenges that you did *not* complete, which of the following describe why you did not complete them? Please select all that apply.

[ ] I could not think of anything that completed the challenge.
[ ] I found the challenge too easy.
[ ] I found the challenge too difficult.
[ ] I found the challenge boring.
[ ] I had already decided what I was going to eat that day.
[ ] I did not see the challenge.
[ ] I did not have time to complete the challenge.
[ ] I forgot about the challenge.
[ ] I completed the challenge, but forgot to take a picture of my food.

Other: ____________

Question 9.
On a scale from 1 (strongly disagree) to 7 (strongly agree), please rate how much you agree with each of the following statements:

[ ] I enjoyed receiving random daily challenges.

[ ] I found the daily challenges boring.

[ ] I felt that it was important to complete the daily challenges.

[ ] I only solved the challenges when it was convenient to do so.

[ ] I went out of my way to solve challenges.

[ ] The challenges made me think about my food choices for the day.

[ ] If it were possible, I would like to continue receiving daily challenges.

Question 10.
On a scale from 1 (strongly disagree) to 7 (strongly agree), please rate how much you agree with each of the following statements:

[ ] I often forgot to take pictures of what I ate through the app.

[ ] I found the daily reminder helpful.

[ ] I would have liked to receive more reminders.

[ ] I found it easy to enter a food using the app on the "I am eating" (Record Food) page.

[ ] I found it easy to look through the food I had eaten on the "History" page.

Part 4 of 4: Food Habits

On how many of the last seven days do you think you ate five or more servings of fruits and vegetables? (a serving is about 2-3 cups, check here for how much of various foods is in a cup).

Question 12.

On how many of the last seven days do you think you ate a serving of a high-fat food, such as red meat or full-fat dairy products?

Question 13.

Please describe how much the following questions apply to you (Never/Rarely, Sometimes, Often, Usually/Always):

[ ] I notice when there are subtle flavors in the foods I eat.

[ ] My thoughts tend to wander while I am eating.

[ ] I recognize when food advertisements make me want to eat.

[ ] Before I eat I take a moment to appreciate the colors and smells of my food.

[ ] I think about things I need to do while I am eating.

[ ] I notice when I'm eating from a dish of candy just because it's there.

[ ] I appreciate the way my food looks on my plate.

[ ] I eat so quickly that I don't taste what I'm eating.

[ ] I recognize when I'm eating and not hungry.

Question 14.
Is there anything else about the app, the daily challenges, or the study that you would like us to know?
