# Supplementary material for: Crumbs: Lightweight Daily Food Challenges to Promote Engagement and Mindfulness
Source: Proc SIGCHI Conf Hum Factor Comput Syst. Author manuscript; Available in PMC 2017 May 12. (PMC5428072; doi:10.1145/2858036.2858044)
Supplement: Pre-survey [file NIHMS855557-supplement-Pre-survey.pdf]

Food4Thought Pre-Survey

Thanks again for participating in our study! In this pre-survey, we are going to collect some basic demographic information, previous experiences keeping track of food, and finally walk you through installing the application. This process should take approximately 20 minutes.

Question 1.
On how many of the last seven days do you think you ate five or more servings of fruits and vegetables? (a serving is about 2-3 cups, check here for how much of various foods is in a cup).

Question 2.
On how many of the last seven days do you think you ate a serving of a high-fat food, such as red meat or full-fat dairy products?

Question 3.
Please describe how much the following questions apply to you (Never/Rarely, Sometimes, Often, Usually/Always):

- [ ] I notice when there are subtle flavors in the foods I eat.
- [ ] My thoughts tend to wander while I am eating.
- [ ] I recognize when food advertisements make me want to eat.
- [ ] Before I eat I take a moment to appreciate the colors and smells of my food.
- [ ] I think about things I need to do while I am eating.
- [ ] I notice when I'm eating from a dish of candy just because it's there.
- [ ] I appreciate the way my food looks on my plate.
- [ ] I eat so quickly that I don't taste what I'm eating.
- [ ] I recognize when I'm eating and not hungry.

Experiences

For the following questions, please provide detail in your responses. We are trying to understand your goals and experiences as much as possible.

Question 4.
Please describe any exercise or health related goals that you have, such as weight maintenance/loss or increasing/maintaining physical activity.

Question 5.
Are you currently on a diet or a fitness regimen? If so, please describe that regimen and for how long you have been on it.

Question 6.
Have you ever tracked your food before with paper or an application (mobile, tablet, desktop)? If so, please explain which method, how long you tracked, and when and why you stopped (or if you are currently tracking your food).

Question 7.
On average, how often do you check Facebook?

- ◯ More than 10 times per day
- ◯ Between 10 and 5 times per day
- ◯ Between 5 and 2 times per day
- ◯ Once per day
- ◯ Once every few days
- ◯ Once per week
- ◯ Less than once per week

Question 8.
On average, how often do you post to Facebook, either on your own timeline or someone else's?

- More than 10 times per day
- Between 10 and 5 times per day
- Between 5 and 2 times per day
- Once per day
- Once every few days
- Once per week
- Less than once per week

Question 9.
In a typical week, which of these do you usually use to access Facebook?

- iPhone
- Tablet
- Computer (Laptop or Desktop)
- Other:
